# Consumers' experiences and values in conventional and alternative medicine paradigms: a problem detection study (PDS)

**DOI:** 10.1186/1472-6882-12-39

**Published:** 2012-04-10

**Authors:** Lynne Emmerton, Jasmina Fejzic, Susan E Tett

**Affiliations:** 1 The University of Queensland, School of Pharmacy, Brisbane, QLD 4072, Australia; 2 Curtin University, School of Pharmacy, Perth, WA 6845, Australia; 3 Griffith University, School of Pharmacy, Gold Coast, QLD 4222, Australia

## Abstract

**Background:**

This study explored consumer perceptions of complementary and alternative medicine (CAM) and relationships with CAM and conventional medicine practitioners.

A problem detection study (PDS) was used. The qualitative component to develop the questionnaire used a CAM consumer focus group to explore conventional and CAM paradigms in healthcare. 32 key issues, seven main themes, informed the questionnaire (the quantitative PDS component - 36 statements explored using five-point Likert scales.)

**Results:**

Of 300 questionnaires distributed (Brisbane, Australia), 83 consumers responded. Results indicated that consumers felt empowered by using CAM and they reported positive relationships with CAM practitioners. The perception was that CAM were used most effectively as long-term therapy (63% agreement), but that conventional medicines would be the best choice for emergency treatment (81% agreement). A majority (65%) reported that doctors appeared uncomfortable about consumers' visits to CAM practitioners. Most consumers (72%) believed that relationships with and between health practitioners could be enhanced by improved communication. It was agreed that information sharing between consumers and healthcare practitioners is important, and reported that "enough" information is shared between CAM practitioners and consumers. Consumers felt comfortable discussing their medicines with pharmacists, general practitioners and CAM practitioners, but felt most comfortable with their CAM practitioners.

**Conclusions:**

This PDS has emphasized the perceived importance of open communication between consumers, CAM and conventional providers, and has exposed areas where CAM consumers perceive that issues exist across the CAM and conventional medicine paradigms. There is a lot of information which is perceived as not being shared at present and there are issues of discomfort and distrust which require resolution to develop concordant relationships in healthcare. Further research should be based on optimisation of information sharing, spanning both conventional and CAM fields of healthcare, due to both the relevance of concordance principles within CAM modalities and the widespread use of CAM by consumers.

## Background

The use of, and beliefs in, complementary and alternative medicine (CAM) *versus *conventional therapies can be culturally determined, sometimes ignoring individual consumers' preferences, rights and needs [1,2]. CAM is a term used for treatments which are not considered part of conventional medicine, including herbal medicines, acupuncture and others. However, conventional and CAM services are being integrated by some types of healthcare practitioners [3-5]. This includes many pharmacists who supply CAM treatments and who are in a position to promote quality use of these medicines, in accordance with the National Medicines Policy in Australia [6,7].

Bringing CAM into the mainstream is viewed by some as a way of reinstating focus on the consumer - the person, not the disease [8,9]. This is consistent with another trend - the increasing focus on 'concordance' in relationships between healthcare practitioners and consumers in the conventional healthcare model [10]. Concordance is based on negotiation between partners in a therapeutic relationship (consumer and healthcare practitioners), its strength being respect for the consumer's agenda and the creation of openness in the relationship [11-14]. CAM is increasingly a reality in healthcare, and one aspect in which concordant relationships may be particularly valuable is around integration of the CAM and conventional medicine paradigms [15-19].

Despite the growing body of literature surrounding concordance, there is a lack of published research exploring concordance in relation to CAM. Research to date on the opinions of healthcare practitioners and consumers has historically been concerned mainly with what *healthcare practitioners *want to know about the relationships between consumers and healthcare practitioner, and their perceptions of the issues consumers were facing in relation to both conventional and CAM treatment modalities [5,17,20-23]. A need for more research about concordance and CAM focusing on the *consumer perspective *is evident.

The aim of the present study was to explore the perceptions and understanding of self-selected CAM consumers on concordance and experiences with conventional medicine practitioners (general practitioners (GPs) and pharmacists) and CAM practitioners. This paper explores a group of self-selected CAM consumer perspectives - their views, perceptions, beliefs and experiences in relation to concordance, CAM and conventional medicines use.

## Methods

Problem-Detection Study (PDS) methods were applied; these originated in marketing and advertising research, and are gaining acceptance in many areas of social health research [24-28]. This method combines qualitative data collection (often using focus groups or interviews) to explore the meanings that people ascribe to their situations and their perceptions of significance, and then quantitative data collection (usually a more widely-administered questionnaire) for numerical data, often used for comparative analysis between different groups [29-31]. The concept of problem identification arising from differing perceptions of the same issue/statement is very useful for future intervention design and evaluation. The principle behind this PDS was to investigate similarities and differences between consumers' ratings of issues relating to CAM and concordance compared to those rated by healthcare practitioners in a previous study, reported elsewhere [10].

This study was granted ethical approval by the Human Research Ethics Committee (number 2005/6) of the School of Pharmacy, The University of Queensland (JF was a postgraduate student at the time). Focus group participants gave written consent, and implied consent was given by participants choosing to complete the questionnaire. All participants could withdraw at any time without consequences, and all data were de-identified.

### Focus group with consumers of CAM

A focus group was conducted with CAM consumers to gather information on participants' thoughts and experiences in an environment that provided an opportunity for participants to freely share their thoughts and ideas on a wide range of issues [31-34]. The CAM consumers were recruited through a written advertisement in three health-food stores, three CAM practices, and three pharmacies in inner-city Brisbane, Australia, over a period of one month. These sites enabled purposive sampling for the purposes of a focused discussion. The inclusion criteria were that the participants could recall using a CAM (very openly defined as 'natural' or complementary medicine bought from a pharmacy, health food store or supermarket), were conversant in English, and were aged at least 18 years. This recruitment method was a combination of convenience and purposive sampling to ensure that the participants were likely to be capable of providing answers within the topic being researched [31,32]. Nine CAM consumers (seven females and two males) were recruited for the focus group, which is comparable with other studies [31-34]. The facilitator of the focus group (LE) encouraged participation and maintained relevance of the discussion. A researcher (JF) was present as an observer and note-taker. Simple starter ('seed') questions, derived from concordance, CAM, and literature relating to focus group research, were formulated in a non-leading and non-suggestive way to ensure that they did not unduly influence the participants' own agendas and opinions [35]. The focus group was recorded and later transcribed *verbatim *by a researcher (JF). The transcript was thematically analysed manually into the key issues by a researcher (JF) and checked independently by the co-researchers (LE, ST) to minimise subjectivity of interpretation. Thirty-two key issues were identified by manual thematic analysis from this process in combination with interviews (10 general practitioners) and focus groups (9 pharmacists) with health professionals (as detailed elsewhere [10]).The identified issues from the consumer focus group (in conjunction with those from the health professional groups, reported elsewhere [10]) were then thematically analysed and summarised manually through a systematic process of data reduction into seven themes by a researcher (JF) [30,31]. No particular number of themes was sought in this analysis. The accuracy of this data reduction was also checked independently by researchers LE and ST to minimise personal interpretation bias [30,31]. These themes were used to develop the questionnaire (below), as described previously [10]. A single CAM consumer focus group was used as data were also collected from the other groups to extract the themes and develop the statements. In total sufficient data were obtained for the extraction and theme development, the purpose of this initial qualitative stage of the PDS.

### Quantitative data collection phase

The quantitative component of the PDS was undertaken via a self-completed questionnaire by the relevant population; this approach mirrored other PDS reports [24-28]. The questionnaire development was informed by the focus group data (from the seven themes developed as described above). Thirty-six statements (corresponding to the seven themes, given in Table legends) (Tables 1, 2, 3, 4, 5, 6 and 7) were developed and a five-point Likert scale applied: 'strongly agree', 'agree', 'neutral', 'disagree', and 'strongly disagree' (with a separate 'not applicable/don't know' option). The seven themes were described through several statements each until the researchers felt that the statements sufficiently and comprehensively described the themes. It was not the intention of the researchers to have equal numbers of statements for each theme, but rather for the themes to be sufficiently outlined in the statements (e.g. the number of statements corresponding to the themes ranges from 3 to 8 per theme).

**Table 1:** Communication and Information Sharing (Theme 1)

Component Statements	Agreement (%)	Neutral (%)	Disagreement (%)	Total (n)
I am encouraged to share the same type of personal health information with my doctor as with my CAM practitioner.	56	5	39	82

I lose confidence in the success of my treatment if I cannot spend enough time in consultation with my healthcare practitioners (conventional and/or CAM).	83	7	10	81

I am given enough information about benefits of my CAM treatment to feel confident about the treatment(s).	76	18	6	82

I am given enough information about risks of my CAM treatment to be confident about the treatment(s).	66	20	14	80

I am given enough information about benefits of my conventional treatment to feel confident about the treatment(s).	32	19	49	83

I am given enough information about risks of my conventional treatment to feel confident about the treatment(s).	45	13	42	82

I am satisfied with the amount of information exchange between me and my conventional health practitioners at present.	37	17	46	83

I am satisfied with the amount of information exchange between me and my CAM practitioners at present.	79	11	10	82

**Table 2:** Harmonious partnerships through understanding individuals' agendas (Theme 2)

Component Statements	Agreement (%)	Neutral (%)	Disagreement (%)	Total (n)
I feel that my choice to use CAM is respected by all health professionals involved in my care.	18	15	67	79

I feel better acknowledged by my CAM practitioner than by my medical doctor.	58	21	21	81

My partnership(s) with my medical doctor(s) empowers me as a consumer more than my partnership(s) with my CAM practitioner(s).	6	27	67	79

My partnership(s) with my pharmacist(s) empowers me as a consumer more than my partnership(s) with my CAM practitioner(s).	8	20	72	80

My partnership(s) with my pharmacist(s) empowers me as a consumer more than my partnership(s) with my medical doctor(s).	20	23	57	81

**Table 3:** Consumer empowerment through accurate/timely information (Theme 3)

Component Statements	Agreement (%)	Neutral (%)	Disagreement (%)	Total (n)
My healthcare practitioners (conventional and/or CAM) have all the necessary information needed to make decisions about my treatment without my direct involvement.	11	7	82	83

I prefer making treatment choices by myself when I receive enough information on the treatment from my health practitioners (conventional and/or CAM).	82	16	2	82

My medical doctor encourages my input into treatment decisions more than my CAM practitioner.	5	20	75	77

Consumers who use CAM are more empowered in their treatment than the consumers who use solely conventional medicines.	77	15	8	82

I would like to take more responsibility for my treatment by getting more involved in every aspect of it (lifestyle and drug treatment).	84	12	4	81

**Table 4:** Empirical beliefs about efficacy (Theme 4)

Component Statements	Agreement (%)	Neutral (%)	Disagreement (%)	Total (n)
Being informed about the treatment is not important if the treatment is working for me.	15	9	76	81

CAM medicines are effective because they have been used for hundreds of years.	58	26	16	76

The Australian Government only allows sale of CAM medicines that have been shown to be effective.	24	35	41	71

**Table 5:** Beliefs about acute as distinct from chronic therapy (Theme 5)

Component Statements	Agreement (%)	Neutral (%)	Disagreement (%)	Total (n)
The most effective use of CAM is as long-term therapy.	63	19	18	78

CAM are best used to prevent disease rather than treat disease.	35	16	49	80

Conventional medicines are the best choice for emergency treatment.	80	16	4	80

**Table 6:** Continuum of conventional to complementary and alternative healthcare (Theme 6)

Component Statements	Agreement (%)	Neutral (%)	Disagreement (%)	Total (n)
Medical doctors feel comfortable with their patients seeing CAM practitioners.	8	27	65	78

Pharmacists are knowledgeable about both conventional and CAM medicines.	18	17	65	77

CAM practitioners are sufficiently trained to recognise when CAM are not enough and conventional medicine needs to be used.	80	11	9	79

There is no need for my CAM practitioners and conventional practitioners to communicate, since the treatments they provide are different.	18	10	72	81

**Table 7:** Concordance (Theme 7)

Component Statements	Agreement (%)	Neutral (%)	Disagreement (%)	Total (n)
I feel more satisfied with my partnership with my CAM practitioner than with my partnership with my pharmacist.	76	10	14	79

I feel more satisfied with my partnership with my CAM practitioner than with my partnership with my medical doctor.	59	22	19	82

I feel more satisfied with my partnership with my medical doctor than with my partnership with my pharmacist.	44	32	24	80

I feel more comfortable discussing my concerns about side effects with my CAM practitioner than with my doctor.	52	22	26	81

I feel more comfortable telling my medical doctor that I am not happy with taking a medicine they prescribed than I do telling the same to my CAM practitioner.	10	16	74	81

I feel comfortable discussing with my pharmacist all of the issues I might have with my medicines.	57	22	21	81

I feel comfortable discussing with my medical doctor all of the issues I might have with my medicines.	72	10	18	83

I feel comfortable discussing with my CAM practitioner all of the issues I might have with my medicines.	81	16	3	80

Wording was tailored to consumers, the statements were randomised, and the questionnaires were anonymous and coded. Pilot testing for face validity involved five consumers. The survey proper was distributed over three months (determined by diminishing returns) via CAM practitioners' waiting rooms, community pharmacies, and health-food stores in Brisbane, Australia. The questionnaire was collected by/given to consumers to complete and return (postage paid). The inclusion criteria were that the consumers could recall ever having used a CAM or could recall having visited a CAM practitioner (again, this was not rigidly defined for them but left open as 'natural' or complementary medicines or naturopaths, herbalists or other complementary and alternative medicine (CAM) practitioners). This study applied purposive and convenience sampling methods to ensure that the participants were likely to be capable of providing answers within the topic being researched [30,31]. It was not the aim of this explorative research to provide data generalisable to the whole population, but rather to examine the data relating to particular populations of respondents interested in the topic [30,31]. A target sample size of 100-150 consumers and anticipated response rate of less than 50% required the distribution of 300 questionnaires. Due to the anonymous nature of the questionnaire and the modes of distribution, there was no follow-up of non-respondent consumers, and no incentives were provided to respondents. A self-addressed postage paid envelope was attached to each questionnaire to facilitate return [31].

The data analysis, described in detail previously [10], involved complete data screening during and after data entry, random checking of the data entry accuracy, analysis of the demographic (categorical) data, and descriptive analysis of the 36 statements (medians and frequencies of response options). Identified issues were then compared, also comparing to group data already published [10], and common trends noted. The findings below report the consumer perspective, including a short discussion contextualising the consumer perspectives against the key findings from the three groups of health professionals previously surveyed (pharmacists, GPs, CAM practitioners [10]).

## Results

A 28% response (83 of 300 questionnaires distributed) was obtained for the consumer participants. More females (58) than males (25) responded. The majority of the consumers were middle-aged (47% aged 35-54 years, n = 47; 21 consumers were 34 or under and 15 were older than 55).

Tables 1, 2, 3, 4, 5, 6, and 7 represent the responses to each statement from the consumer group. The statements are classified under the corresponding seven themes and shown as worded for the consumers. 'Don't know/not applicable' responses are excluded from the tabulated results, and for ease of interpretation and taking into account the sample size, the 'strongly agree/agree' and the 'strongly disagree/disagree' categories have been collapsed into single categories 'agreement' and 'disagreement', respectively.

There was general agreement with the importance of information sharing between consumers and healthcare practitioners, and a reported belief that "enough" information is shared between CAM practitioners and consumers (Table 1).

There was a reported belief amongst the consumers (67%) that their choice to use CAM was not respected by health professionals, however they felt better acknowledged by their CAM practitioner than by their medical doctor. Overall, the consumers felt most empowered by their relationship with their CAM practitioner(s) and least empowered by their relationship with their pharmacist(s) (Table 2).

Consumers demonstrated willingness (84% in agreement) to be actively involved in their healthcare, as evident in the statement "*I would like to take more responsibility for my treatment by getting more involved in every aspect of it (lifestyle and drug treatment)*", and felt that they were quite encouraged to do so by the CAM practitioners (Table 3). As CAM consumers, they considered themselves to be more empowered than people who were not using CAM.

Most consumers also wished to be informed about effective treatments, and believed in historical effectiveness of CAM. Some consumers (24%) believed that the Australian government only allowed the sale of *effective *CAM, although the majority did not report an opinion (Table 4).

The opinion was expressed that CAM are used most effectively as long-term therapy (63% in agreement), but less agreement that this would be for treatment as well as for prevention (35% in agreement), but the opinion also was that conventional medicines would still be the best choice for emergency treatment (81% in agreement) (Table 5). The majority of consumer respondents (65%) reported that they perceived that conventional doctors felt uncomfortable about consumers' visits to CAM practitioners. They also indicated that they thought CAM practitioners had a high level of knowledge about CAM and pharmacists did not. Most consumers (72%) believed that CAM and conventional practitioners need open channels of communication (Table 6).

Consumers, on the whole, felt that they were more satisfied with their partnerships with CAM practitioners than their partnerships with their medical doctors, and they were least satisfied with their partnerships with their pharmacists. Consumers felt comfortable discussing their medicines with all three groups of health practitioners, but were most comfortable with their CAM practitioners (Table 7).

## Discussion

This PDS adds to the field by exploring perceptions about concordance, CAM and conventional medicine use and the views of self-selected CAM users about CAM and conventional health providers. It is then possible to compare and contrast these data with the same information collected from conventional healthcare providers [10] and consumers of conventional medicines to detect important areas of similarities and differences in perceptions between groups. This detection of contrasts and similarities can then assist in developing future innovations to bridge gaps in understanding and enhance holistic healthcare.

The majority of CAM consumers surveyed in this PDS were of the view that there was insufficient information exchanged between consumers and conventional medicine practitioners, which contrasted with their majority view that there was sufficient mutual information exchange between consumers and CAM practitioners. The surveyed consumers' perceptions of not being acknowledged by the GP can potentially have serious ramifications, since this can also cause consumers not to disclose their CAM use [23,36]. Literature supports the notion that the use of CAM is not sufficiently discussed between doctors and their patients during consultation [37-39], with disclosure of CAM use to doctors by their patients occurring in around 60-70% of cases [40,41], while an Australian study reported CAM use being reported to the medical practitioner in only fewer than half of the cases [42]. It has been reported that consumers who experience a more participatory style of consultation with conventional practitioners tend to be more likely to also reveal that they are using CAM [36]. It has also been reported that even when the patients were willing to discuss CAM with their doctor, the doctor still may not feel comfortable about it [17]. In addition, interestingly, it has been shown that doctors tend to be uncomfortable about collaborating with CAM practitioners even when they work in the same hospital teams [43]. On the other side of the spectrum, an American study indicated that some family doctors preferred to call themselves 'holistic' and align themselves with CAM in order to attract more consumers [44]. Further, it has been found that doctors sometimes tend to report high acceptance of CAM, even though their knowledge of them was, self-reportedly, quite poor, creating a potentially dangerous situation [45]. Conversely, some CAM practitioners are indeed willing to work with conventional medicine practitioners in seeking a patient-centred integrated health system [3]. There is probably still quite a way to go to achieve true collaboration and open communication in interprofessional relationships in healthcare [46]. Our previous study also indicated that all practitioners (conventional and CAM) valued communication, but that their understanding of each other's roles tended to be dubious and inadequate, adversely affecting the necessary first step in their relationship building [10].

The surveyed consumers, on the whole, thought that their partnerships with CAM practitioners were more empowering to consumers than consumers' partnerships with pharmacists and doctors. Similarly, a German study reported that patients using CAM appeared to be more closely involved in the decision-making process, reported more satisfaction with treatments, and felt more acknowledged than conventionally-treated patients [47]. The finding that our sample of consumers reported more empowering relationships with doctors than with pharmacists was surprising, given repeated data supporting pharmacists as one of the most trusted professions [48]. Perhaps empowerment is best generated through more formal consultations (doctors' surgery, naturopath clinic), or through remunerated consultations.

Most respondents felt that they, CAM consumers, were more empowered than non-CAM consumers. The CAM consumer responders tended to value longer consultations with their CAM practitioner(s), the 'whole person' approach, mutual respect and sharing decisions, all of which encourages their perception of being empowered [49]. Further, it has been reported that the majority of patients who prefer a patient-centred approach also preferred CAM use, and *vice versa *[50]. However, another study found that there was no statistically significant difference between users and non-users of CAM with respect to their preferred role in healthcare [51].

A study applying patient profiling in consumers' decision making on CAM use suggested that it is possible that there are not just alternative therapies but 'alternative patients' - people who are simply more inclined to use CAM [52]. Identifying these patients early could help healthcare practitioners to optimise their advice on treatment options and to approach these patients in a suitable way [52]. This early identification could also be utilised to open the channels of communication and increase the chance of achieving mutually-acceptable partnerships (concordance) between consumers and all of their healthcare providers.

It seemed that some surveyed consumers had poor knowledge about the regulation of CAM treatments available in Australia, stating that CAM available in Australia have proven effectiveness. This is not true as CAM currently do not have to have proven effectiveness in order to be available on the Australian market [53]. It is unclear how this consumer perception affects the degree to which they use CAM, as it has been reported that even when there is no scientific evidence on the effectiveness of CAM, consumers still tend to continue to use them [54].

The use of 'mixed methods' (i.e. qualitative then quantitative methods), combined in this PDS, served to firstly extract themes of relevance, and then to formulate these as specific issues for quantitative comparison with previous responses by health professionals [10] and for within-group analysis (i.e. spread of responses amongst this sample of consumers). One focus group was held to inform development of the PDS questionnaire from the self-selected CAM consumers' perspective, and this provided a rich source of generated data (in conjunction with previous focus groups with other health professional groups [10]), sufficient for the extraction and development of the themes.

The importance of health-related information sharing between consumers and healthcare practitioners was emphasized by this consumer focus group and questionnaire. It should be noted that the intention of this study was not to explore the nature of the consumers' relationship with a specific type of CAM practitioner, how many CAM practitioners consumers regularly consulted, nor did it examine their individual experiences with CAM use.

The limitations of this study included the low response rate to the questionnaire, and the small number of participants in the CAM consumer focus group. The data from the focus group, however, were used in conjunction with previously collected data [10] to develop the resulting questionnaire. This single focus group was not used in isolation. The findings from the survey, the quantitative part of the PDS reported here, are not intended to be extrapolated to other consumer populations, as we recognise that the self-selection was biased towards users of CAM, and that the response rate gave a relatively small sample size. From previous PDS methodology reports, [24-28] it is clear that the response rates to mailed-back surveys of consumers are expected to be low or variable [55-58]. Some of the documented techniques used to improve response rates were applied in the present study, for example, endorsement by a university, the use of a concise questionnaire, and administration with a self-addressed envelope for return. Even using these techniques still only resulted in a 28% response, which is low. This does, however, give a window of insight into this self-selected group of CAM consumers and is valuable for exploring differences between this group and the perceptions of health professionals, which could lead to recommendations about which differences in perceptions need to be attended to in order to achieve the goal of therapeutic alliances ('concordance'). Care must be taken not to extrapolate too widely from these findings. The respondents were CAM consumers and may have an inherent bias towards CAM medicines and possibly CAM practitioners (and likely different opinions on conventional medicines when compared to non-CAM consumers). Interestingly, Australian census data, referred to by Xue et al., indicate that approximately 69% of the population have used CAM in the preceding 12 months and 44% have visited a CAM practitioner [42]. Further, in Australia, the estimated number of visits to a CAM practitioner, cited by Xue et al., is almost identical to the estimated number of visits to the GP annually (69 million) [42].

The focus of this overall analysis was the consumer, and the sub-analyses compared and contrasted their perceptions and opinions on conventional medicine and CAM; however, this PDS approach is also useful when compared to other data, collected from other groups, to determine differences and similarities between groups. Thus it is useful to briefly consider the main points of agreement and disagreement of the three previously-surveyed health professional groups (general practitioners, pharmacists, and CAM practitioners) in relation to the consumers' perceived roles [10].

Interestingly, there were numerous statements on which the majority of participants across *all four groups *(including this study's CAM consumers' responses) *agreed*. Some of these statements have potentially significant ramifications in terms of their effect on the ways in which relationships are established and advanced in healthcare now and into the future. The fact that the majority of all three practitioner groups and CAM consumers agreed on these statements makes them quite informative in terms of current shared beliefs and experiences in the healthcare system:

• Consumers share the same type of personal health information with all of their healthcare practitioners, but insufficient information is exchanged between consumers and conventional healthcare practitioners;

• Consumers would lose confidence in treatment if they did not spend enough time with their healthcare practitioners;

• Consumers prefer making treatment choices by themselves when they receive enough information on treatments from all healthcare practitioners;

• Consumers' direct involvement in treatment decisions is always valued and they would like to take more responsibility for their treatments by becoming more involved in their treatment decision making;

• It is important to inform consumers on their treatment whether it is working for them or not;

• Medical doctors are not comfortable with their patients seeing CAM practitioners, but there is a need for CAM and conventional practitioners to communicate;

• Consumers' choice to use CAM is not respected by all health practitioners involved in their care.

It is encouraging to note the goodwill in the perceptions of the four groups of participants towards further development of sound relationships. However, some inconsistency was evident - a majority belief across all four groups suggested the need for CAM practitioners and conventional practitioners to communicate, but at the same time that medical doctors are not comfortable with their patients seeing CAM practitioners. Our PDS, especially the comparisons of the quantitative responses to the statements from the self-selected CAM consumer group with data from the conventional and CAM providers, gives good indications for future areas of research. Endeavours to move acceptance and understanding about CAM use by consumers into the realm of conventional medicine can support these areas of mutual agreement and intervene to dispel areas of misunderstanding. Identification of these issues and the underlying relationships, potentially leading to concordance relationships, both for consumers and conventional medicine practitioners or CAM practitioners, are very valuable, providing a starting point for further investigation.

## Conclusions

The concept of concordance is based on forming a therapeutic alliance between partners in healthcare. With an assumption of respect for the consumer's agenda and the creation of an atmosphere of openness in the therapeutic relationship, consumers and healthcare practitioners can form their relationships on the basis of reality rather than misunderstanding, distrust and concealment. Perceptions of superiority of either CAM or conventional medicine can diminish mutual respect between healthcare practitioners, and, directly or indirectly, influence consumers' perceptions, communication and behaviour. This PDS has emphasized the importance of open communication between consumers, CAM and conventional providers, and exposed areas where CAM consumers perceive that issues exist. There is a lot of information which is perceived as not being shared at present and there are issues of discomfort and distrust which require resolution.

It would be warranted to elucidate what exactly are the perceived 'positives' of the CAM paradigm that are appealing to the consumer, and to apply these in the development of concordant relationships with all health practitioners. Our research suggests that the perceived empowerment and open relationships between consumers and CAM practitioners may be significant issues warranting further attention. Most of the potentially problematic issues identified in this study could be minimised by enhancement of communication between various health practitioners, and between consumers and health practitioners. Hence, further research in the field of concordance should be based on *optimisation of information sharing *among the partners and our research gives indications of some problem areas, identified by one or more groups. It is recommended that such research spans *both conventional and CAM fields *of healthcare, due to the relevance of concordance principles within CAM modalities, and the widespread use of CAM by consumers. Consequently, further research evaluating the health systems and potential interventions enhancing communication from the consumers' perspective would be useful. It is hoped that future researchers will keep using creative approaches to finding the balance between CAM and conventional aspects of healthcare in order to optimise relationships between all stakeholders and improve health outcomes.

## Competing interests

The authors declare that they have no competing interests.

## Authors' contributions

JF, LE and ST conceived the study and participated in the design and execution of the research. JF did the initial transcribing and thematic analysis, independently checked by LE and ST. All authors participated in questionnaire design. JF completed the initial data analysis and manuscript preparation, with supervision from LE and ST, as part of her PhD thesis. All authors read and approved the final manuscript.

## Note

The work was carried out at The University of Queensland, School of Pharmacy, Brisbane, QLD 4072, Australia.

## Pre-publication history

The pre-publication history for this paper can be accessed here:

http://www.biomedcentral.com/1472-6882/12/39/prepub
